# Repeated mechanical damage enhanced *Aquilaria sinensis* resistance to *Heortia vitessoides* through jasmonic acid

**DOI:** 10.3389/fpls.2023.1183002

**Published:** 2023-08-08

**Authors:** Yingying Chen, Shenghua Liang, Shuyao Wang, Baocai Li, Kun Wang, Yongjin Zhu, Risheng Yang, Xin Hao, Zhuoying Yang, Yingbai Shen, Rihong Jiang, Kaixiang Li

**Affiliations:** ^1^ Guangxi Key Laboratory of Special Non-wood Forests Cultivation and Utilization, Guangxi Xylophyta Spices Research Center of Engineering Technology, Illicium and Cinnamomum Engineering Technology Research Center of National Forestry and Grassland Administration, Guangxi Forestry Research Institute, Nanning, China; ^2^ College of Biological Sciences and Technology, Beijing Forestry University, Beijing, China; ^3^ National Engineering Laboratory of Animal Breeding, College of Animal Science and Technology, China Agriculture University, Beijing, China

**Keywords:** *Aquilaria sinensis*, defense response, herbivory wounding, mechanical damage, *Heortia vitessoides*

## Abstract

**Introduction:**

The leaf-chewing pest *Heortia vitessoides* severely threatens the growth and development of *Aquilaria sinensis*. In our previous study, we found that mechanical damage (MD) to stem enhanced *A. sinensis* sapling resistance to *H. vitessoides* larvae.

**Methods:**

To reveal the defense mechanisms underlying this observation, we analyzed the types and contents of volatile organic compounds (VOCs), phytohormone contents, and expression of phytohormone-related genes in response to MD and herbivory wounding(HW).

**Results:**

Here, we identified several VOCs, such as the pesticides fenobucarb and 2,4-di-tert-butylphenol, in mature leaf (ML) of MD-treated plants. Compared with salicylic acid (SA) or the ethylene (ET) pathway, jasmonic acid (JA) content and JA-related genes were more strongly upregulated. Interestingly, we found a dramatic difference between JA-related upstream and downstream genes expression in YL and ML, which confirmed that JA-Ile accumulation in MD-ML and HW-ML could be derived from local damaged site.

**Discussion:**

Taken together, we provide evidence that the JA pathway plays a dominant role in the *A. sinensis* response to MD and HW.

## Introduction

Incense tree (*Aquilaria sinensis* (Lour.) Spreng.), an evergreen tropical tree in the Thymelaeaceae, is well known for its agarwood, which has great economic and medicinal value and is often used in traditional medicine, religious ceremonies, daily cosmetics, and other industries. Numerous studies have investigated the mechanisms underlying the formation of agarwood and offered ways to develop efficient techniques for its promotion (Wang et al., 2022; Zhang et al., 2022). These studies are made more difficult by the exposure of *A. sinensis* trees to a wide range of harsh environmental stimuli, especially herbivory wounding (HW). In our previous investigation, we demonstrated that insect pests severely threaten *A. sinensis* growth, for example, infestation with 3- to 5-instar-stage larvae of the moth *Heortia vitessoides* (Lepidoptera: Pyralidae) frequently leads to large areas of trees with no leaves in artificial *A. sinensis* forests, which seriously limits plant growth and can even result in the death of the trees.

*Heortia vitessoides* Moore (Lepidoptera: Crambidae) is distributed across southern China and some regions of southeast Asia. As a typical oligophagous insect, *H. vitessoides* larvae feed on several species of the genera *Aquilaria* and *Rhus*, although they feed solely on the leaves of *A. sinensis* in China (Su, 1994; Chen et al., 2011). In one artificial *A. sinensis* forest, over 90% of trees were predated by the larvae (Sha et al., 2018), with a single tree commonly harboring thousands of larvae. Such a high insect density often resulted in leafless trees after a few days, leaving the forest bare (Su, 1994; Sha et al., 2018). We previously observed this phenomenon in our investigation in Qinzhou, Guangxi, China, between March and August 2021. In nature, *H. vitessoides* imagos prefer YL for oviposition, potentially attracted by the aldehydes and esters they release (Yan et al., 2019). Indeed, our observations showed that larvae preferred to consume YL, rather than ML, and will not consume ML until all the YL are eaten (**Supplementary Figure S1**). We therefore hypothesized that this preference might not entirely result from the thicker waxy layer of ML, but also from their greater accumulation of specific metabolites compared to YL. These substances may reduce leaf palatability and may even be toxic to larvae. Under laboratory conditions, larval feeding resulted in the death of nearly 30% of saplings. Although pesticides temporarily alleviate the adverse effects of these insect pests, the negative consequences of excessive pesticides should not be ignored. Natural predatory or parasitic enemies have also been used to limit *H. vitessoides* densities (Qiao et al., 2012; Yan et al., 2019; Zhou et al., 2019). Despite these studies, how *A. sinensis* responds to herbivorous insects is largely unknown, and its defensive system, formed over long-term co-evolution with their predators, has been neglected.

In the past few decades, extensive scientific attention has been placed on the defense responses of a wide variety of plant species. The plant defense response can be defined as a series of specific internal metabolic changes and external structural changes that improve their ability to survive exposure to insect or pathogen attacks. These responses involve the perception of external stimuli, signal transduction, the regulation of defense gene expression, the accumulation of biologically active compounds, and finally the realization of the defense effect (Zhang et al., 2019). Plants emit specific blends of volatile organic compounds (VOCs) in response to HW or mechanical damage (MD). Herbivory-induced and wound-induced plant VOCs not only play an important role in plant communication, but also are used as defense substances to directly repel pests or attract their natural enemies (parasitoids and predators), and can even directly produce toxic or intoxicating effects on pests (Kikuta et al., 2011; Turlings, 2018). Several C6 aldehydes, alcohols, and their esters, so-called green leaf volatiles (GLVs), attract natural enemies of primary attacking pests. In addition, other volatiles, such as terpenoids, are also thought to play a role in plant defense responses. These volatiles are released by both local and systemic leaves within several hours of HW. Numerous studies have shown that the release of herbivory-induced plant VOCs is orchestrated by a network of phytohormones, including jasmonic acid (JA), ethylene (ET), and salicylic acid (SA) (Mujiono et al., 2020; Hu et al., 2021). Their signaling pathways actively crosstalk with those of other phytohormones through individual, synergistic, or antagonistic effects, leading to increased or decreased resistance to multiple stresses. Interestingly, the JA and SA pathways are considered to be mutually antagonistic, generally, SA mediates responses to biotrophic pathogens and piercing or sucking insects, while JA mediates the plant response to necrotrophic pathogens and chewing insects (Xu et al., 2021). Whether ET plays a positive or negative role in plant defense responses remains unclear, for example, exogenous ET inhibits VOC production in rice (*Oryza sativa* L.) (Mujiono et al., 2020), while a JA-mediated increase in VOCs can be induced by an application of the ET precursor 1-aminocyclopropane-1-carboxylate (ACC) in lima beans (*Phaseolus lunatus* L.) (Horiuchi et al., 2001). To date, the mechanisms by which phytohormones precisely regulate defense responses in *A. sinensis* to HW remain unclear. In the present study, we explored the mechanisms underlying the *A. sinensis* defense responses induced by *H. vitessoides* feeding and MD, providing new insights to facilitate the development of efficient and environmentally friendly strategies to control this insect pest.

## Materials and methods

### Plant materials

Ripe *A. sinensis* fruits were collected in June or July of 2020-2022. It takes about 1–2 days for the fruit shells to crack open naturally under cool and ventilated conditions. After the fruit shells cracked, the seeds were collected and soaked in 0.1% (w/v) KMnO_4_ for 20 min, then rinsed with water 3–5 times before being soaked in water for 6 h. Fine sand, pre-disinfected with 0.5% (w/v) KMnO_4_ for 4 h, was laid on a 1.2 × 2-m sand bed at a thickness of 12 cm. The above prepared seeds were laid flat on the sand bed and covered with a thin layer of sand. Saplings with 3–4 true leaves were moved into plastic pots (13 cm diameter × 16 cm tall) containing yellow soil (pH 4.5–6.0). These saplings were then grown in a growth chamber under a 16-h light (29°C)/8-h dark (26°C) photoperiod, with 60% relative humidity.

### Insect larvae materials

Leaves with attached *H. vitessoides* eggs were collected from *A. sinensis* trees in Beiliu, Guangxi, China. These leaves were placed on YL of *A. sinensis* seedlings in a growth chamber under a 16-h light/8-h dark photoperiod, and newly hatched larvae fed on seedling leaves. During this period, any adverse factors that interfered with their growth were eliminated until they were tested in the present study. Newly hatched larva are too sensitive to environmental stimuli to perform a choice experiment to investigate their feeding preferences. Thus, one-week-old larvae were used in the choice experiment, while two- to three-day-old larvae were used to observe their growth patterns.

### Herbivory wounding treatment

For HW treatment, *H. vitessoides* larvae were starved for 6 h before being used in the experiment. They were then placed on YL of *A. sinensis* and timed until they began to consume leaves. The larvae used for this experiment were restricted to YL rather than ML. In general, larvae were removed when leaf loss reached 30%–40%. Then, samples were harvested and immediately frozen in liquid nitrogen and stored for detection of target genes expression, phytohormones, or volatile metabolites. It was noteworthy that the number of larvae used in qRT-PCR experiment was not restricted. The shorter time to obtain the sample, the more larvae were needed. And the timing started when the larvae start feeding on the leaves.

### Mechanical damage treatment

To perform the repeated MD treatment, a scalpel was used to slice the stem of *A. sinensis* saplings from the base to the apical site at 1-cm intervals. A damage depth of 0.5–0.8 mm was necessary to ensure that both the phloem and xylem on one side of the stem were wounded, but without affecting normal sapling growth. We define the time from scratch initiation to obtaining sample or using saplings as the period of MD treatment. These saplings could be directly harvested at different MD point or used to perform the next step treatment (herbivory wounding, HW).

### Combination of mechanical damage and herbivory wounding treatment

The combination of MD and HW (MH) was achieved by first subjecting saplings to the above MD method. After 24 h, one-week-old starved larvae were placed on YL for the HW feeding assay. Once feeding damage area of young leaf reached by 30%~40%, we removed all larvae.

### Phenidone and exogenous methyl jasmonate treatments

To reinforce the essential role of JA in *A. sinensis* defense against *H. vitessoides* larvae, phenidone (0.5 mM), an inhibitor of JA biosynthesis and exogenous MeJA (5 μM) were used in in this study (Chen et al., 2020). About 200 mL of 0.5 mM phenidone was applied into the saplings pots with 2 day intervals. The control individuals were treated with purified water. Twelve hours later, the above pretreated saplings were subjected to MD for 24 h, named as PM-treated saplings.

Then, we sprayed MeJA (5 μM) on the leaf of PM-treated saplings, which was regarded as PMM-treated group. Healthy plants with consistent growth and without any damage were used as the control group. The control group was spayed by distilled water containing ethyl alcohol (one in 25,000, v/v). About 5 mL MeJA liquid was used on each sapling every time. Then, theses saplings were used to carry out the larvae choice experiment, larvae growth assay, feeding damage area assay, detection of JAs content. Additionally, the samples for JAs content detection were obtained at HW 5 h (herbivory wounding for 5 h), MD 5 h(mechanical damage for 24h), MH 5 h(MD-24h following HW-5h), Phe 12 h(0.5mM phenidone for 12 h), PMH (combination of Phe 12 h and MH 5 h), PMMH (Phe+MeJA+MH), respectively.

### *H. vitessoides* larvae choice experiment

Four-month-old healthy saplings were used in this experiment. A scalpel was used to scratch the saplings’ stems, which served as the MD group. Saplings without any treatment were used as the control group. The two groups of saplings were then placed in two separate rooms for 24 h. To avoid disturbing the insects’ feeding behavior, these experiments were carried out in a studio with uniform light. The two groups of saplings were arranged in a cross, and larvae were placed in the center to record their behavior. At least one hundred larvae were tested in this experiment. After 6 h, the number of larvae was counted on every seedling.

### *H. vitessoides* larvae growth assay

To investigate the effect of MD, PM or PMM pretreated with *A. sinensis* saplings on larval growth, ten-month-old saplings and One-instar-stage larvae were used in a growth assay. After a 24-h treatment with MD, PM, or PMM, fifteen larvae were placed on YL of each plant. Five days later, the lengths and weights of larvae or feeding damage area were determined, respectively. A control group of larvae was fed with saplings without any damage.

### Detection of volatile metabolites

Fresh plant materials were harvested, weighed, then immediately frozen in liquid nitrogen and stored at –80°C until needed. The samples were ground into a powder in liquid nitrogen, and about 1 g of powder was subjected to sample extraction. Automatic headspace solid-phase micro-extraction (HS-SPME) (Agilent, Palo Alto, CA, USA) was used to extract the samples. The extracts were analyzed using a GC apparatus (model 8890B, Agilent Technologies, Santa Clara, California, USA) and a 8000D mass spectrometer (Agilent Technologies) for the identification and quantification of VOCs.

An unsupervised PCA was performed on the scaled unit variance data using the statistics function prcomp within R (www.r-project.org). Hierarchical cluster analysis (HCA) of the samples and metabolites is presented as heatmaps with dendrograms, while Pearson’s correlation coefficients (PCC) between samples were calculated using the cor function in R and presented as heatmaps. Both HCA and PCC were carried out using the R package p heatmap. For HCA, the normalized signal intensities of metabolites (unit variance scaling) were visualized as a color spectrum. Significantly differentially regulated metabolites between the groups were determined with a VIP ≥ 1 and an absolute Log2FC (fold-change) ≥ 1. VIP values were extracted from the OPLS-DA results generated using the R package MetaboAnalystR, which also contain score plots and permutation plots. The data were Log_2_-transformed and mean-centered before OPLS-DA.

### Analysis of fenobucarb insecticidal activity against *H. vitessoides* larvae

Fenobucarb, a known pesticide (Tada and Hatakeyama, 2000), was induced by MD. This compound was therefore studied to determine whether it had insecticidal activity against *H. vitessoides* larvae. Four- to five-instar *H. vitessoides* larvae were cultured in a chamber at 26–28°C. A pesticide containing 80% fenobucarb (the other 20% merely a solvent) was obtained from Yunju (Guangxi, China), and was diluted 500, 1000, and 1500 times. A 1-mL aliquot of each dilution was transferred into a 9-cm Petri dish containing filter paper, to which 10 healthy larvae with uniform growth were added. The time required for 50% mortality (half lethal time, LT50) of the larvae was recorded. A Petri dish containing 1 mL distilled water served as the control group.

### Measurement of phytohormone contents

Fresh leaves or stem with different treatments were harvested, weighed, immediately frozen in liquid nitrogen, and stored at –80°C until needed. To extract phytohormones, 50-mg (fresh weight) samples were ground into powder in liquid nitrogen then extracted using 1 mL methanol/water/formic acid (15:4:1, v/v/v). The combined extracts were evaporated until dry under a nitrogen gas stream, reconstituted in 100 μL 80% methanol (v/v), and filtered through a 0.22-μm filter for LC-MS analysis. OPDA, JA, JA-Ile, SA, and ACC levels in the plants were determined by MetWare (http://www.metware.cn/) based on an AB Sciex (Framingham, Massachusetts, USA) QTRAP 6500 LC-MS/MS platform.

### RT-qPCR assay

The relative expression levels of various phytohormone biosynthesis genes were tested in YL and ML of four-month-old saplings at 0 h, 1 h, 2 h, 4 h, 8 h, 12 h, and 24 h after treatment with HW, MD, or MH. Total RNA was extracted from the samples using an RNA extraction kit (CWBio, Beijing, China), and first-strand cDNA was generated using a reverse transcriptase kit (Monad, Suzhou, China). The relative expression of several key genes in the JA, SA, and ET biosynthesis pathways was detected (Lv et al., 2019), including LOX (lipoxygenase), AOC (allene oxide cyclase), AOS (allene oxide synthase), OPR (12-oxo-phytodienoic acid reductase), JAR (JASMONOYL ISOLEUCINE CONJUGATE SYNTHASE), JAT (JA transporter), a key enzyme for SA biosynthesis: ICS (isochorismate synthase), PBS (*avr*PphB SUSCEPTIBLE) and a key enzyme for ET biosynthesis: 1-aminocyclopropane-1-carboxylic acid oxidase (ACO). We searched for candidate genes in the *A. sinensis* genome (Ding et al., 2020) encoding putative JA transporters (JATs), JAR, OPR, and PBS. Finally, We identified two putative *JAT* genes, one JAR gene, one OPR gene, and one PBS gene from the genome data, which were named *AsJAT1*, *AsJAT2*, *AsOPR, AsJAR and AsPBS*, respectively. *AsGADPH* was used as the reference gene, as described previously (Sun et al., 2020). Detailed primer information is provided in **Supplementary Table S1**. The putative *AsJAT1*, *AsJAT2, AsOPR, AsJAR and AsPBS* sequences are provided in **Supplementary Table S2**. qPCR was performed with an Applied Biosystems 8500 fast real-time PCR system (Thermo Fisher Scientific, Waltham, Massachusetts, USA) using a QantiNova SYBR Green PCR kit (Qiagen, Hilden, Germany), and the 2^−∆∆Ct^ method was used to calculate relative gene expression levels.

## Results

### Mechanical damage enhanced the *A. sinensis* resistance to *H. vitessoides*

In the early stage, we pretreated four-month-old *A. sinensis* saplings with MD for 24 h before subjecting them to an insect choice test. We observed that 88% of larvae are more likely to choose control plants without MD treatment, whereas only 20% of larvae selected MD plants, 3% of larvae did not choose any plant during the course of the experiment, which we marked as an invalid selection (I.S.) (**Supplementary Figure S2**). These results indicate that MD might help *A. sinensis* repel *H. vitessoides* larvae.

**Figure 1:**
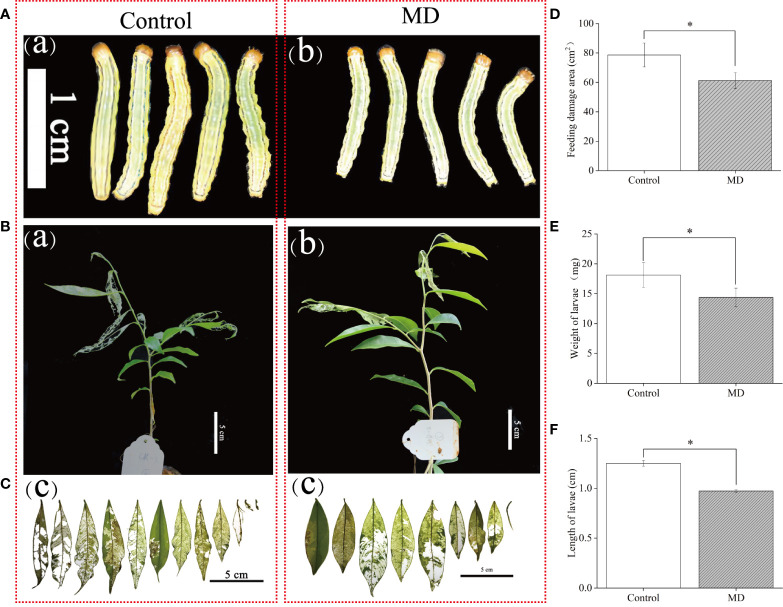
Effect of mechanical damage on *A. sinensis* defense against *H. vitessoides* larvae. **(A)** Phenotype of *H. vitessoides* larvae reared with MD-treated or unwounded saplings for 5 days. The white scale bar represented 1 cm. **(B)** Representative photograph of *A. sinensis* saplings with or without a MD pretreatment after *H. vitessoides* larvae feeding for 5 days. Scale bars, 5 cm. **(C)** representative photograph of *A. sinensis* leaves after larvae feeding. Scale bars, 5 cm. **(D)** Feeding damage area of control or MD-treated *A. sinensis*. Scale bars, 5 cm. Length **(E)** and weight **(F)** of *H. vitessoides* larvae reared on control or MD saplings. Different lowercase letters in photograph A-C represent the phenotype of larvae or plants with different treatments. Data are means ± SE. Asterisks indicate significant differences between MD and Control individuals (*P < 0.05; Student’s *t*-test). Three replicates were conducted.

To verify whether MD improves *A. sinensis* resistance to *H. vitessoides*, larvae growth experiment were performed. we fed two- or three-day-old *H. vitessoides* larvae with MD plants for 5 days, while the control group was fed with control saplings not exposed to MD. As shown in **Figure 1**, we observed that the size of larvae reared with control-plants (**Figure 1A**) was bigger than that fed on MD-plants (**Figures 1A, B**). The upper YL was first consumed both in MD- and control plants (**Figure 1B**). Separately, the leaves of control plants were severely damaged (**Figure 1B-a**). Compared with control-group, feeding damage area of MD-plants (61.2 cm^2^) decreased by ~23%. (**Figures 1C, D**). The growth of larvae fed with MD-treated saplings was significantly inhibited (**Figures 1A, B**). Compared to the control group, MD-group larvae were both shorter and lighter (**Figures 1A, E, F**). These results suggest that MD of the stem could initiated the *A. sinensis* resistance to *H. vitessoides* larvae through repressing larvae growth. ML may exhibit a stronger defense response than YL.

At the end of the above experiment, we placed the saplings damaged by HW alone or the combination of MD and HW in controlled growth conditions (16-h light at 29°C/8-h dark at 26°C, with 60% relative humidity). Four months later, MD-HW treated saplings had completely recovered their growth vigor with a 100% survival rate, while HW saplings showed a lower survival rate (83%) and were smaller (dwarfed) (**Supplementary Figure S3**). Thus, MD pre-treatment was not only conducive to the *A. sinensis* defense against *H. vitessoides* larvae, but also benefited the recovery of plant growth after insect feeding.

### Effects of MD on volatile metabolites in ML

**Figure 2:**
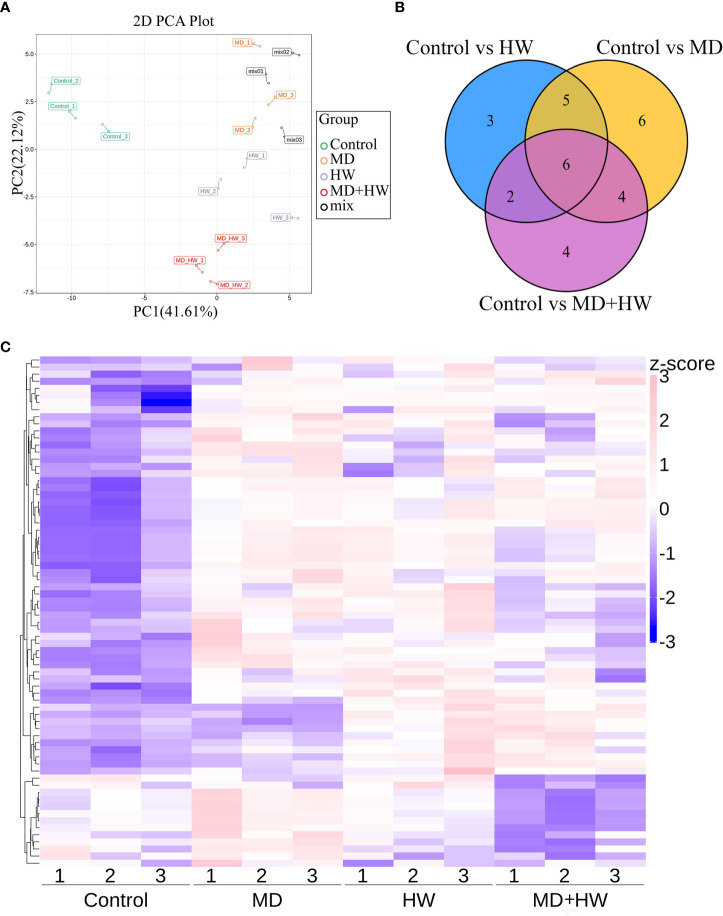
Metabolome profiling of *A. sinensis* mature leaves following a MD, HW, or MH treatment. **(A)** Principal component analysis (PCA) score plot of ML along the first component (PC1) and second component (PC2). **(B)** Venn diagram showing the overlap between differentially abundant metabolites identified in treated leaves relative to control leaves. **(C)** Hierarchical clustering and correlation analysis of the metabolites in ML following MD, HW, or MH treatment.

To investigate the volatile metabolites primed by MD, HW, or the combination of MD and HW (MH group), we performed an automatic headspace solid-phase micro-extraction (HS-SPME) to extract volatile samples, followed by gas chromatography (GC)–mass spectrometry (MS) to determine the volatile metabolites in mature *A. sinensis* leaves exposed to different treatments. As shown in **Figure 2**, we detected a total of 81 metabolites in mature *A. sinensis* leaves, most of which being ketones, aldehydes, alcohols, phenols, and esters. Through a principal component analysis (PCA) of these metabolites, we divided our 12 samples into four different clusters corresponding to the different treatments, namely the control group, MD group, insect feeding (HW) group, and the MD + insect feeding (MH) group. In the model, 41.6% and 22.1% of the total variance could be explained by PC1 and PC2, respectively (**Figure 2A**). Consistently, a hierarchical clustering and correlation analysis showed that the leaf metabolites are strongly affected by the different treatments (**Figure 2C**). The relative contents of volatile metabolites in ML significantly accumulated following HW, MD, and MH treatments. The metabolites from the MH and HW groups clustered into a secondary branch, suggesting that the metabolite changes induced by MH are more similar to those induced by the HW group than by the MD group (**Figure 2C**). Compared to control plants, HW, MD, and MH caused the specific accumulation of three, six, and four metabolites, respectively, in ML (**Figure 2B**).

To identify the variable metabolites among all treated plants relative to control plants, we generated an OPLS-DA (Orthogonal Projections to Latent Structures Discriminant Analysis) model. We determined that 81 metabolites differentially accumulate in ML of treated plants. Among them, we detected 16, 21, and 16 differentially accumulated metabolites (VIP [variable influence in project] ≥ 1, *p*-value < 0.05, fold-change ≥ 2 or fold-change ≤ 0.5) for the comparisons of the control group and HW, MD, and MH plants, respectively. The number of upregulated and downregulated metabolites in each comparison are listed in **Table 1**. In HW plants, 16 differentially accumulated metabolites reached higher levels than in control plants (detailed in **Table 2**). We detected three specific metabolites induced by HW in ML: 2-methoxy-phenol (MW0029), n-propyl ether 2-propylphenol (MW0068), and phthalic acid, butyl 2-pentyl ester (MW0082) (**Supplementary Figure S4**; **Table 2**). Relative to the comparison between control and HW plants, we observed a greater variation in abundant metabolites for the comparison between control and MD plants. Among the 81 metabolites detected in MD-treated plants, 21 metabolites were significantly differentially accumulated. Eleven common substances increased in ML from both MD and HW plants, while six metabolites only significantly increased in MD plants (**Supplementary Figure S5**; **Figure 2B**; **Tables 1**, **2**). These results provided evidence that about 50% of the differentially accumulated metabolites induced by MD are not induced by HW, suggesting that *A. sinensis* distinguishes biotic stress (insect feeding) and abiotic stress (MD). We detected six specific compounds induced by MD in ML: fenobucarb (MW0048), α-ionone (MW0053), 2(3H)-benzoxazolone (MW0060), 5-pentyl-1,3-benzenediol (MW0065), 4,6-bis(1,1-dimethylethyl)-2-ethyl-4-methyl-4H-1,3,2-dioxaborin (MW0066), and (R)-5,6,8,8a-tetrahydro-4,4,8a-trimethyl-2(4H)-benzofuranone (MW0068). Among these six specific metabolites, fenobucarb was reported to be a carbamate insecticide (Tada and Hatakeyama, 2000). We used a pesticide based on fenobucarb in a touch-kill test, during which we established that the time required to kill half of the test larvae (LT_50_) was about 30 min (**Supplementary Table S3**). It is worth noting that the most abundant MD-elicited compounds are pyrazines, quinolines, phenols, and esters, for example, 4-ethylamino-1-butanol (MW0031) was 6.1 times higher in MD leaves than in control leaves, while (2S,4aS,5R,8aR)-2,5-dipropyldecahydroquinoline (MW0058) was 5.9 times higher in the MD group than in the control group (detailed in **Table 2**). These substances may play important roles in the *A. sinensis* defense response to *H. vitessoides* larvae.

**Table 1:** Number of differentially accumulated metabolites in pairwise comparisons between control *A. sinensis* trees and those subjected to HW, MD, or MH treatment.

Group name	All sig. Diff.	down regulated	up regulated
Control_vs_HW	16	0	16
Control_vs_MD	21	0	21
Control_vs_MD_HW	16	2	14
HW_vs_MD	0	0	0
HW_vs_MD_HW	3	3	0
MD_vs_MD_HW	8	8	1

Metabolites with a significantly increased or decreased accumulation are listed separately for each comparison.

**Table 2:** Differentially accumulated metabolites in the ML of *A. sinensis* following the MD, HW, or MH treatments.

Index	RT	Compounds	Control vs HW		Control vs MD		Control vs MH	
			Fold Change	Type	Fold Change	Type	Fold Change	Type
MW0006	5.5188	Hexanal					0.4	down
MW0019	11.9016	o-Cymene					0.4	down
MW0021	12.1810	Methanesulfonyl chloride					2.6	up
MW0029	13.8196	2-methoxy-Phenol	2.1	up				
MW0031	14.2431	4-Ethylamino-1-butanol	5.4	up	6.1	up	3.1	up
MW0038	16.2455	5-(acetyloxy)-2-Pentanone	5.5	up	5.2	up	6.3	up
MW0038	16.8121	2-Methoxy-5-methylphenol					2.4	up
MW0039	16.9105	Methyl 5(3)-methyl-4-hydroxy-3(5)-pyrazolecarboxylate	2.0	up			2.1	up
MW0040	16.9156	Orotic acid	2.1	up	2.0	up	2.2	up
MW0042	18.0828	4-Hydroxy-3-nitropyridine N-oxide	2.1	up			2.2	up
MW0043	18.3859	3-(acetyloxymethyl)-2,2,4-trimethyl-Cyclohexanol			2.0	up	2.1	up
MW0044	18.3885	4,5-Dimethyl-2-isobutyloxazole			2.0	up	2.2	up
MW0045	18.6120	N-trifluoroacetyl-Methiopropamine			2.1	up	2.4	up
MW0048	20.1249	tetrahydro-6-propyl-2H-Pyran-2-one	4.8	up	5.3	up	4.4	up
MW0048	20.4500	Fenobucarb			2.3	up		
MW0050	21.6846	Benzene, 1,4-dimethyl-2,5-bis(1-methylethyl)-	4.1	up	4.2	up	3.3	up
MW0053	23.2248	.alpha.-Ionone			2.3	up		
MW0056	24.5493	4-(Diethylamino)benzoic acid	5.3	up	5.1	up	3.5	up
MW0058	24.6150	(2S,4aS,5R,8aR)-2,5-Dipropyldecahydroquinoline	5.6	up	5.9	up		
MW0058	24.6204	4,4a,5,6,8,8,9,9a-octahydro-10,10-dimethyl-1,4-Methano-1H-cyclohepta[d]pyridazine	5.8	up	5.8	up		
MW0059	24.6385	dimethyl ether 4-tert-Butylcatechol	5.8	up	5.6	up		
MW0060	25.1389	2(3H)-Benzoxazolone			2.0	up		
MW0062	25.2856	2,4-Di-tert-butylphenol	2.8	up	3.4	up		
MW0065	25.5936	5-pentyl-1,3-Benzenediol			2.1	up		
MW0066	25.5944	4,6-bis(1,1-dimethylethyl)-2-ethyl-4-methyl-4H-1,3,2-Dioxaborin			2.1	up		
MW0068	25.9280	(R)-5,6,8,8a-tetrahydro-4,4,8a-trimethyl-2(4H)-Benzofuranone			2.8	up		
MW0068	26.5889	n-propyl ether2-Propylphenol	2.6	up				
MW0069	28.5501	Hexadecane	2.4	up	2.2	up		
MW0081	30.1522	Pentadecanal			2.1	up	4.0	up
MW0082	33.1684	Phthalic acid, butyl 2-pentyl ester	2.2	up				

The metabolite index number and retention time (RT), as well as their abundance in HW, MD, and MH plants relative to the control are provided. The fold-change and increase or decrease of differentially accumulated metabolites are listed separately for each comparison.

In MH plants, we detected 16 differentially abundant metabolites, comprising 14 upregulated compounds and two downregulated compounds (**Table 2**; **Supplementary Figures S6A, B**). A cluster analysis showed that the two significantly decreased substances (hexanal (MW0006) and O-cymene (MW0019), **Table 2**) cluster together, as did the 14 increased substances but in a different portion of the plot (**Table 2**; **Supplementary Figure S6C**).

### Effect of HW on gene expression and phytohormones in the *A. sinensis* leaves

To explore the *A. sinensis* defense mechanisms against insect feeding, we considered three phytohormone signaling pathways (JA, SA, and ET). We detected the relative expression of typical genes that participate in JA, SA, or ET biosynthesis (Koornneef and Pieterse, 2008; Torrens-Spence et al., 2019), such as those encoding key enzymes in JA biosynthesis: LOX, AOC, AOS, OPR, JAR or JAT, two key enzyme for SA biosynthesis: ICS and PBS, and a key enzyme for ET biosynthesis: ACO.

**Figure 3:**
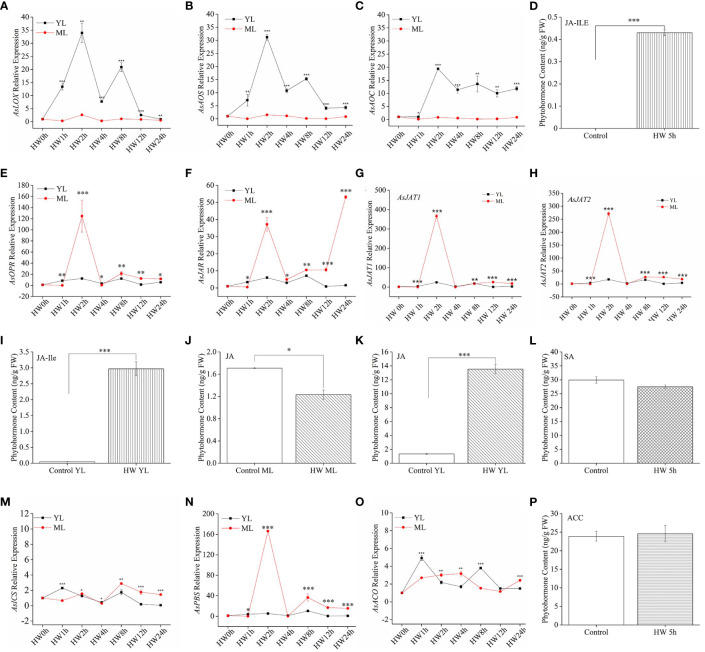
JA-Ile, SA, and ACC contents and expression of their related genes in *A. sinensis* induced by HW. **(A–C)**, **(E–H)** JA-related genes (*AsLOX*, *AsAOS,AsAOC, AsOPR, AsJAR, AsJAT1, AsJAT2*) expression in YL and ML after HW. **(D)**, **(I–K)** JA-Ile and JA content in YL and ML after HW 5 h. **(L)** SA content in ML after HW 5 h. **(M, N)** Relative expression of the SA-related gene *AsICS* and *AsPBS* in YL and ML after HW. **(O)** Relative expression of the ET-related gene *AsACO* in YL and ML after HW. **(P)** Content of ACC, the direct precursor of ET, in ML after HW 5 h. Four- to ten-month-old plants were used in these experiments. Insect feeding was limited to YL. All samples for gene expression analyses were harvested at 0 h, 1 h, 2 h, 4 h, 8 h, 12 h, and 24 h after HW. Asterisks in **(A–C), (E–H)** and **(M–O)** indicate significant differences between YL and ML (*P < 0.05; **P < 0.01; ***P < 0.001; Student’s t-test). Asterisks in **(D)**, **(I–K)** indicate significant differences in ML under different treatments. Data are means ± SE. Different lowercase letters indicate significant difference (*P < 0.05; **P < 0.01; ***P < 0.001; Student’s t-test). Three replicates were carried out. At least ten saplings were used in each replicate.

For JA pathway genes, the expression levels of *AsLOX*, *AsAOS*, and *AsAOC* in YL subjected to HW were significantly higher than in ML without HW (**Figures 3A–C**). From 2 h to 8 h after HW, the highest expression of the three JA biosynthesis genes exceeded a 30-fold change in YL relative to the control. Despite of low expression of *AsLOX*, *AsAOS*, and *AsAOC* in ML, a significant JA-Ile increase was detected both in ML (**Figure 3D**) and YL (**Figure 3E**) induced by HW. Then, we detected JA content in YL and ML in *A. sinensis* with or without HW. As shown in **Figures 3J, K**, the content of JA in HW-group ML significantly decreased, whereas that in YL increased. Interestingly, downstream genes *AsOPR, AsJAR* and *AsJATs* exhibited dramatic up-expression in ML rather than YL(**Figures 3E–H**). For instance, the relative expression of *AsJAT1* and *AsJAT2* in ML was 366- and 281-fold greater at HW-2 h than that in the control-ML, respectively. In YL, these two genes showed a 24- and 18-fold upregulation, respectively (**Figures 3G, H**), suggesting the elevation of JA-Ile in ML might be transmitted from local damaged YL, or derived from key intermediates. These results indicated a specific mechanism in *A. sinensis* priming systematic defensive response.

Despite of two major metabolic routes for SA biosynthesis exist in plants, ICS and PAL (phenylalanine ammonolyase) pathway (Mishra and Baek, 2021). Only *AsICS* and *AsPBS* involving in ICS pathway were considered in this present study. Because ICS route has been regarded as the major pathway contributing to more than 90% of SA synthesis in plant (Rekhter et al., 2019). In **Figure 3M**, we observed *AsICS* expression with a less 4-fold change in ML. But *AsPBS* exhibited great higher up-expression in ML than in YL **Figure 3N**. The above two genes in ML were more sensitive to HW treatment. However, we did not obtain an obvious change on the content of SA between HW-treated ML and control-ML (**Figure 3L**), suggesting SA could not be the dominant pathway in *A. sinensis* defense against larvae feeding.

Additionally, we also detected a key gene for ET production (*AsACO*) and its important precursor ACC. In our test period, the expression of AsACO only showed ~5 fold change at HW 1 h (**Figure 3O**). Although significant difference existed between YL and ML, these up-regulated expression by HW were much lower than JA-related genes (**Figures 3A–C, E–H**). Predictably, the content of ACC in ML from HW-plants did not exhibited any difference from control-ML (**Figure 3P**).

### Effect of MH and MD on gene expression and phytohormones in the *A. sinensis* leaves

**Figure 4:**
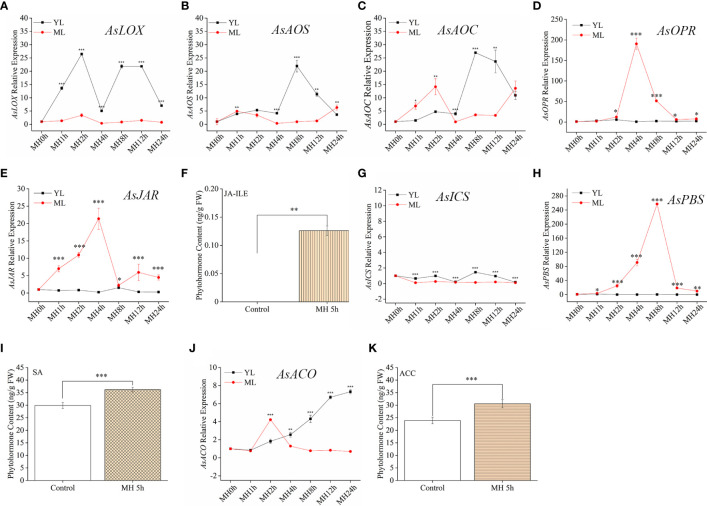
JA-Ile, SA, and ACC contents and expression of their related genes induced by a combination of MD and HW (MH). The combination of MD and HW (MH) was achieved by first subjecting saplings to MD. After 24 h, starved larvae were placed on YL for the HW feeding assay. After 0 h, 1 h, 2 h, 4 h, 8 h, 12 h, and 24 h, larvae were moved away. Then samples were harvested at MH 0 h, MH 1 h, MH 4 h, MH 8 h, MH 12 h, and MH 24 h, respectively. **(A–E)** JA-related genes expression in YL and ML after MH treatment. **(F)** JA-Ile content in ML after MH 5 h. **(G, H)** Relative expression of the SA-related gene *AsICS* and *As PBS* in YL and ML after MH. **(I)** SA content in ML after MH 5 h. **(J)** Relative expression of the ET-related gene *AsACO* in YL and ML after MH. **(K)** ACC content in ML after MH 5 h. Asterisks in **(A–E, G, H, J)** indicate significant differences between YL and ML (*P < 0.05; **P < 0.01; ***P < 0.001; Student’s t-test). Asterisks in **( F, I, K)** indicate significant differences in ML under different treatments. Data are means ± SE.

Although JA pathway showed a stronger sensitive to HW, we should not use a single stress to predict the plant resistance in coping with complex multiple stresses. To better illustrate the mechanism of *A. sinensis* dealing with MH stress, it was sill necessary to detect the variation of related phytohormones and their signaling pathway. in this section, JA-Ile, SA, ACC and gene related to these compounds synthesis were also measured. MH plants showed a similar expression pattern to HW plants in terms of their phytohormone-associated genes. *AsLOX*, *AsAOS, AsAOC* (**Figures 4A–C**) exhibited much stronger up-regulation in YL, whereas *AsOPR* and *AsJAR* showed higher expression in ML (**Figures 4D, E**). Similarly, JA-Ile elevation was also observed in ML with MH 5 h (**Figure 4F**). Unlikely to HW, MH triggered SA significantly accumulated in ML, along with up-expression of *AsPBS* (**Figure 4H**). Despite of *AsICS* expression in YL differed from ML, both of their expression level were much lower than JA-related genes and *AsPBS* (**Figure 4G**). We found an interesting results that *AsACO* up-expression gradually increase in YL, along with the extension of MH treatment period (**Figure 4J**). The content of ACC significantly increased in ML (**Figure 4K**). These results were different to that in plants treated with HW, revealing a complex mechanism in *A. sinensis* coping with MH.

**Figure 5:**
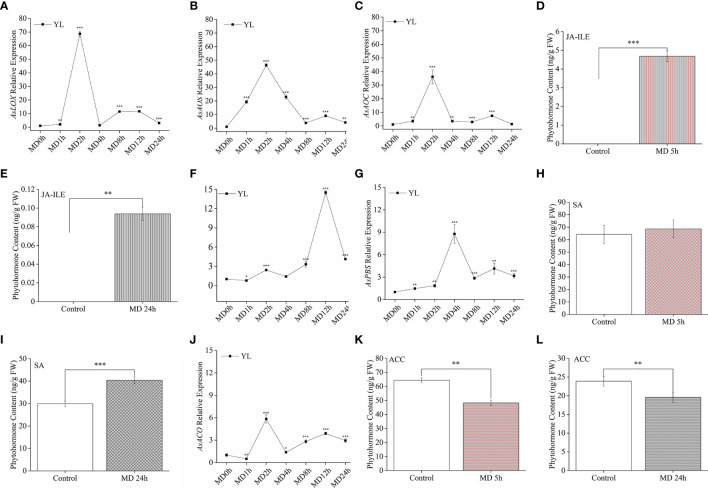
JA-Ile, SA, and ACC contents and expression of their related genes in YL induced by MD. **(A–C)** JA-related gene expression in YL after MD. **(D, E)** JA-Ile content in ML after MD 5 h and MD 24 h. **(F, G)** Relative expression of the SA-related gene *AsICS* and *AsPBS* in YL after MD. **(H, I)** SA content in ML after MD 5 h and MD 24 h. **(J)** Relative expression of the ET-related gene *AsACO* in YL after MD. **(K–L)** ACC content in ML after MD 5 h and MD 24 h. All samples for gene expression analyses were harvested at 0 h, 1 h, 2 h, 4 h, 8 h, 12 h, and 24 h after MD treatment. Asterisks in **(A–C, F, G, J)**, indicate significant differences at different times compared to the 0 h in YL (*P < 0.05; **P < 0.01; ***P < 0.001; Student’s t-test). Asterisks in **(D, E, H, I, K, L)** indicate significant differences of phytohormones in ML between MD and control group. Data are means ± SE.

Here, we asked how and in which MD pretreatment altered phytohormones response. Since HW treatment was strictly limited on YL, we proceeded to investigate the variation of phytohormones and related genes in YL response to MD. *AsLOX*, *AsAOS*, and *AsAOC* (**Figures 5A–C**) exhibited a much stronger upregulation than *AsICS* (**Figure 5F**), *AsPBS* (**Figure 5G**) or *AsACO* (**Figure 5J**) in YL elicited by MD. The expression levels of *AsLOX* (**Figure 5A**), *AsAOS* (**Figure 5B**), and *AsAOC* (**Figure 5C**) peaked after 2 h, and were up to 36-fold, 46-fold, and 68-fold greater than the control, respectively. These high expression levels began to decrease at 4 h after MD treatment, while *AsICS* (**Figure 5F**) and *AsPBS* (**Figure 5G**) expression still increased slowly. We measured phytohormone contents after MD 5 h and 24 h. Compared to control plants, JA-Ile was elevated in ML at 5 h after MD treatment (**Figure 5D**), but it decreased at24 h (**Figure 5E**). Conversely, SA content was not obviously different at 5 h (**Figure 5H**), but significantly accumulated at 24 h (**Figure 5I**), which may indicate an antagonistic effect between the SA and JA pathways. For the ET pathway, the relative expression of *AsACO* exhibited a similar trend as the JA-related genes, although its expression level was much lower than *AsLOX*, *AsAOS*, or *AsAOC* (**Figure 5J**). Compared with control individuals, ACC content in MD-ML was significantly decreased neither at 5 h nor 24 h (**Figures 5K, L**). These evidence suggested in YL, MD could first activated JA pathway, following SA pathway.

### A central role for the JA pathway in the *A. sinensis* response to MH

In our previous observation, it was found that larvae preferred to consume PM plants (~60% of larvae) than control plants (19% of larvae) (**Supplementary Figure S7**). To verify the effect of phenidone and MeJA on JAs compounds synthesis, ten-month-old *A. sinensis* saplings were treated with HW, MD, MH, Phe, PMH, PMMH, respectively. And then *A. sinensis* leaves (including young and mature leaves) were harvested to detect endogenous level of JA and JA-Ile and OPDA. We found that the level of OPDA, JA, and JA-Ile were both increased by HW and MH, which was consistent with our previous results. Once phenidone was applied to *A. sinensis* saplings before MH treatment, these compounds content were decreased, especially OPDA and JA. However, exogenous application of MeJA (PMMH group) could alleviate the inhibition of phenidone on JAs accumulation to a certain extent (**Supplementary Figure S8**). These results confirmed that phenidone can be used as an inhibitor to limited JA biosynthesis. And exogenous application of MeJA could compensate endogenous level of jasmonic acid in *A. sinensis*.

**Figure 6:**
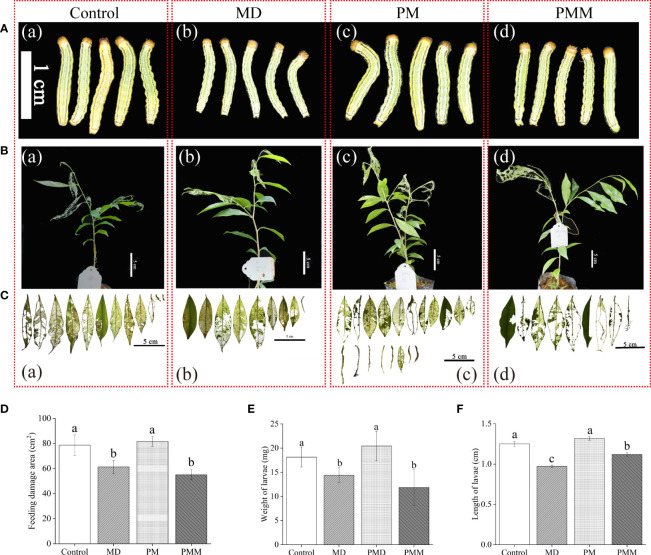
Essential role of MD-induced JA pathway in *A. sinensis* resistance to *H. vitessoides* larvae. One-instar-stage *H. vitessoides* larvae were fed with saplings treated with or without MD, PM, or PMM for 5 days. **(A)** The phenotype of *H. vitessoides* larvae fed with saplings treated with or without different treatments. The white scale bar represented 1 cm. **(B)** Representative photograph of *A. sinensis* saplings with or without MD, PM, or PMM pretreatment after *H. vitessoides* larvae feeding. Scale bars in photograph represented 5 cm. **(C)** Representative photograph of feeding damage area from control or MD-, PM-, PMM-treated saplings. Scale bars in photograph represented 5 cm. **(D–F)** indicated feeding leaf area of *A. sinensis*, larvae length and weight of *H. vitessoides* larvae reared on control or MD-, PM-, PMM-treated saplings, respectively. Different lowercase letters in photograph **(A–C)** represent the phenotype of larvae or plants with different treatments. Different lowercase letters in **(D–F)** indicated significant difference at *P* < 0.05. Data are means ± SE. Three replicates were performed.

To confirm the role of JA in the *A. sinensis* response to MH, defense against *H. vitessoides* larvae, phenidone (0.5 mM) and exogenous MeJA (5 μM) were used in in this study. Then, we evaluated insects resistance of these saplings with different treatments. Compared to the MD group (**Figures 6A-b, E, F**), both body length (**Figures 6A–C, F**) and weight (**Figure 6F**) of PM-group larvae were higher. Meanwhile, feeding damage area of PM-treated plants significantly increased by ~25% comparing with MD-plants (6C-c, 6D). Once the application of MeJA, larvae growth and feeding damage area of leaf were all limited (**Figures 6A–d, B–d, C-c, D–F**), indicating MeJA recovered PM-plants resistance to larvae. These results suggest that MD positively induced a local and systematic resistance in a JA-dependent manner, helping to protect *A. sinensis* from *H. vitessoides* larvae.

**Figure 7:**
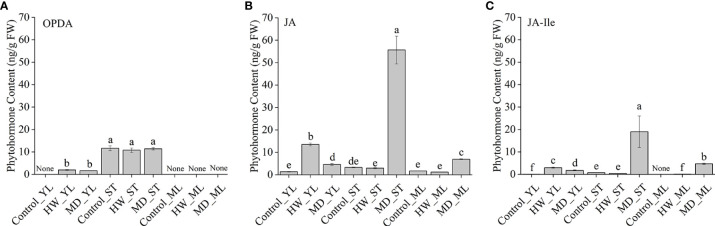
The variation of JAs content in YL, ML and ST (Stem) under HW or MD treatment. Ten-month-old saplings were treated with HW (herbivory wounding for 5 h), MD (mechanical damage for 5 h). The variation of OPDA **(A)**, JA **(B)**, JA-Ile **(C)** content in YL, ML, ST under different treatments. None in A-C represent undetected substance. Each column value represents the mean ± SE (n = 3). Different letters indicate significantly different values at *P* < 0.05.

To clarify how MD enhanced *A. sinensis* resistance to insects through JA pathway, we retested OPDA, JA, and JA-Ile content in YL, ML and ST (the damaged stem). Ten-month-old saplings were treated with HW (herbivory wounding for 5 h), MD (mechanical damage for 5 h), respectively. Interestingly, OPDA remained a high level in *A. sinensis* stem, and its content was not affected by HW or MD. In ML, we did not detected OPDA in all samples. But in YL, an increase of OPDA was induced by HW and MD (**Figure 7A**). JA was detected in all samples. Especially, we observed a dramatic high level of JA in MD-ST, which was about 19 times more than in HW-ST. Meanwhile, JA was significantly increased both in MD-YL and MD-ML(**Figure 7B**). Analogously, JA-Ile also increased by MD and HW treatment, and the highest accumulation was in MD-ST following MD-ML. The content of JA-Ile in MD-ST was 45 and 78 times higher than that of HW-ST and HW-ML, respectively (**Figure 7C**). That may be the reason why MD enhanced *A. sinensis* insects resistance. Notably, JA-Ile was not detected in control-ML. These evidence confirmed that the JA-Ile in ML could be transmitted from stem or derived from intermediate substance.

## Discussion

This study clearly highlights that repeated MD to *A. sinensis* stem is effective in enhancing plant defense against *H. vitessoides* larvae through the JA pathway. We demonstrated that *A. sinensis* responded differently to HW, MD, MH. The growth of *H. vitessoides* larvae was significantly limited by MD, likely because distinct VOCs and systematic resistance depending on JA pathway.

In our previous observation, the threat of *H. vitessoides* on *A. sinensis* is almost year-round in Guangxi (China), especially from March to August. Several researchers have attempted to exploit predators, parasites, or pathogenic bacteria for use against *H. vitessoides*, for instance, the *Trichogramma pintoi* parasitizes 1- to 4-day-old *H. vitessoides* eggs, although it was not effective at targeting 5- to 8-day-old eggs (Yan et al., 2019). Other strategies, such as infection with pathogenic bacteria (Zhao et al., 2019) or attracting insects away using bio-pheromones or certain volatiles (Qiao et al., 2012; Zhou et al., 2019) also play an important role in biological prevention. Pest-killing lamps is used to lure and kill *H. vitessoides* imagos (Qiao et al., 2018b). Other researchers are searching for specific *A. sinensis* germplasm with high resistance to biotic or abiotic stress (Mao et al., 2017), but this approach is hampered by the extremely long breeding cycle of this tree. In the present study, we attempted to shed light on the mechanisms underlying the defense response to larval feeding in *A. sinensis*, providing new insights for pest control in a cost-effective manner and for management during the sapling phase. Interestingly, we determined that *A. sinensis* saplings with repeated stem damage were not predated as heavily by *H. vitessoides* larvae, which preferred to feed on undamaged plants (**Supplementary Figure S2**). This phenomenon suggested that resistance to herbivore wounding might be primed by MD to the stem in this species, which was supported by the extent of larval growth seen in our experiments (**Figure 1**).

Crosstalk between the responses to mechanical wounding and biotic stress has previously been reported, involving some important signaling components: early signaling events, defense-related genes, phytohormone signaling pathways, toxic proteins, and metabolites (Taylor et al., 2004; Zebelo and Maffei, 2015; Deb et al., 2016). In lima bean, volatiles were induced by continuous MD for 30 min, whereas a single MD action did not induce early signaling events or VOC emissions (Bricchi et al., 2010). The transcriptional profiles of Arabidopsis (*Arabidopsis thaliana*) following exposure to abiotic or biotic stresses were previously reported to largely overlap with the expression profiles induced by MD (Walley et al., 2007). In Walley’s study (Walley et al., 2007), 5 min of continuous MD was sufficient for plants to activate defense responses, suggesting that a rapid perception and effective response mechanism exists in plants under continuous mechanical wounding. A rather systematic work was reported by Qiao et al., who investigated the interaction between *A. sinensis* and *H. vitessoides* imagos (Qiao et al., 2012; Qiao et al., 2018a). They identified volatiles induced by insect feeding and leaf-damaged samples, revealing that insect feeding increased green leaf volatile (GLV) contents, which might play a role in imago repulsion. It is worth noting that quantitative and qualitative changes in GLV levels of MD-leaf plants within 8 h were significantly lower than those induced by insects chewing on plants for 1–2 days. The decreased GLVs and increased aldehydes and alcohols in insect-chewed plants meant they eventually became attractive to adults again. (Z)-3-hexenyl acetate was previously reported to be a dominant GLV component with a significant effect on imago attraction (Qiao et al., 2012), however, this effect was not significant in a later study (Qiao et al., 2018b). These studies underscore the complexity of plant responses to environmental stresses, highlighting the need for more evidence to elucidate the mechanisms underlying the *A. sinensis*–*H. vitessoides* interaction. In the present study, a series of esters, aldehydes, phenols, and other substances involved in pesticide production significantly accumulated in ML in response to various wounding treatments (**Table 2**). Several of these compounds are used in fungicides and insecticides, for example, pyridazin (MW0058), a nitrogenous heterocyclic compound, and its derivatives have widely antiinflammatory, antimicrobial, insecticidal and herbicidal activities (Zhou, 2010). The dimethyl ether 4-tert-butylcatechol (MW0059) exhibits a remarkable bactericidal effect and can also be used as an antimicrobial agent (Song et al., 2022). Thus, HW induced the production of defensive compounds (**Supplementary Figure S4**; **Table 2**). About 50% of the differentially abundant metabolites induced by MD were not induced by HW, suggesting that *A. sinensis* indeed distinguished between biotic stress (insect feeding) and abiotic stress (mechanical damage). It was previously reported that hexanal released from young *A. sinensis* leaves plays a role in attracting female *H. vitessoides* imagos (Yan et al., 2019). In our present study, we noticed a lower level for hexanal in MH plants, indicating that MH treatment may weaken this attractive effect (**Supplementary Figure S6**; **Table 2**).

As core signaling components, JA, SA, and ET play essential roles in plant responses to biotic or abiotic stress in synergetic or antagonistic ways (Erb and Reymond, 2019; Yang et al., 2019; Xu et al., 2021). In tomato (*Solanum lycopersicum* L.) plants, wounding promotes the expression of defense genes and activates the accumulation of JA and its associated proteins (Schallera and Oecking, 1999). In the JA biosynthesis pathway, LOX, AOS, and AOC catalyze linolenic acid (LeA) in a step-by-step manner to produce 12-oxophytodienoic acid (OPDA), which is then reduced by OPDA reductase (OPR), and undergoes three rounds of β oxidation before its conversion into JA. JA-Ile, the most bio-active JA molecule, is generated by jasmonoyl amino acid conjugate synthase (JAR), which is required for JA perception and signaling (Fürstenberg-Hägg et al., 2013; Wasternack and Song, 2016). There are two major metabolic routes for SA biosynthesis in plants, ICS and PAL (phenylalanine ammonolyase) pathway (Mishra and Baek, 2021). And ICS has been regarded as the major pathway contributing to more than 90% of SA synthesis (Rekhter et al., 2019). Recently, Wu et al. (2023) found that ^13^C-labeled phenylalanine (^13^C_6_-Phe) was not detected into SA in *Arabidopsis thaliana*. Although they observed the elevation of PAL level in *A. thaliana* upon pathogen infection, ^13^C_6_-SA did not be detected in PAL-overexpressing lines. In this study, we tend to pay attention to PBS (*avr*PphB SUSCEPTIBLE), coding a cytosolic amidotransferase, which catalyzes the conjugation of glutamate to isochorismate (IC) to produce isochorismate-9-glutamate (IC-9-G) (Mishra and Baek, 2021). Then, IC-9-G spontaneously decomposes into SA and 2-hydroxy-acryloyl-N glutamate. However, SA elevation could initiate feedback inhibition on PBS activity (Rekhter et al., 2019), indicating a key role of PBS in modulating SA metabolic pathway. Although AsPBS was up-regulated by HW or MD, SA level in ML did not increase at HW or MD early stage (**Figures 3**, **5**). However, MH triggered SA significantly accumulated in ML, along with up-expression of *AsPBS* (**Figure 4**). More effort should be attempt to reveal the role of PBS in plant response to biotic or abiotic stress in the future.

In *A. sinensis*, JA-Ile, SA, and ACC abundance increased in ML due to stem wounding, and their associated genes (*AsLOX*, *AsAOS*, *AsAOC*, *AsICS*, *AsACS*, and *AsACO*) were strongly upregulated (Lv et al., 2019). In our study, JA-related genes in YL were significantly upregulated by MD (**Figure 5**). We did not detect the expression of the ET pathway gene *AsACS* in YL or ML, suggesting that the expression of *AsACS* described by Lv (2019) may be involved in ET biosynthesis in the stem but not leaves. Additional *ACS* family members should be tested in the future. Given the large proportion of ML in a tree, it is reasonable to believe that ML play an important role in improving insect resistance following MD. Upon *A. sinensis* subjecting to HW, JA biosynthesis was activated at local damaged YL site, such as up-expression of *AsLOX*, *AsAOC, AsAOS*, while the downstream genes (*AsOPR, AsJAR, AsJAT1, AsJAT2*) up-regulated significantly (**Figure 3**). Then, precursor or intermediate increased in ML (**Figure 3**), providing evidence that the increase of JA-Ile in ML might depend on OPR, JAR or JAT. According to recent studies, wound-induced systemic resistance depends on the transmission of mobile wound signals and the accumulation of JA in distant unharmed leaves (Li et al., 2020; Li et al., 2021). Li and colleagues (Li et al., 2021) reported that two JA transporters (AtJAT3/AtJAT4 homo-/hetero-dimers) participate in the long-distance transmission of JA in Arabidopsis. MH plants showed a similar expression pattern to HW, *AsLOX*, *AsAOS, AsAOC* exhibited much stronger up-regulation in YL, whereas *AsOPR* and *AsJAR* showed higher expression in ML (**Figure 4**). Similarly, JA-Ile elevation was also observed in ML (**Figure 4F**). Despite of *AsICS* expression in YL differed from ML, both of their expression level were much lower than JA-related genes and *AsPBS* (**Figure 4G**). These results indicated SA elevation induced by MH could be derived from *AsPBS* activity. And MD pretreatment could promoted this action in an unclear manner to help *A. sinensis* response to HW.

Compared with ET- and SA- pathway, JA pathway was more sensitive to HW. The JA biosynthesis genes (**Figures 3A–C**) exhibited much stronger up-regulation in response to HW than the SA biosynthesis gene *AsICS, AsPBS* (**Figures 3M–N**) or the ET biosynthesis gene *AsACO* (**Figure 3O**). The inhibition of JA biosynthesis abolished the resistance induced by MD (**Figure 6**), confirming the essential role of JA in priming the *A. sinensis* defense response. According to **Figure 7** data, it was clearly that MD induced a dramatic JAs accumulation in ST, YL and ML. We therefore suggested that the JA pathway is likely the dominant pathway in *A. sinensis* in response to *H. vitessoides* wounding.

**Figure 8:**
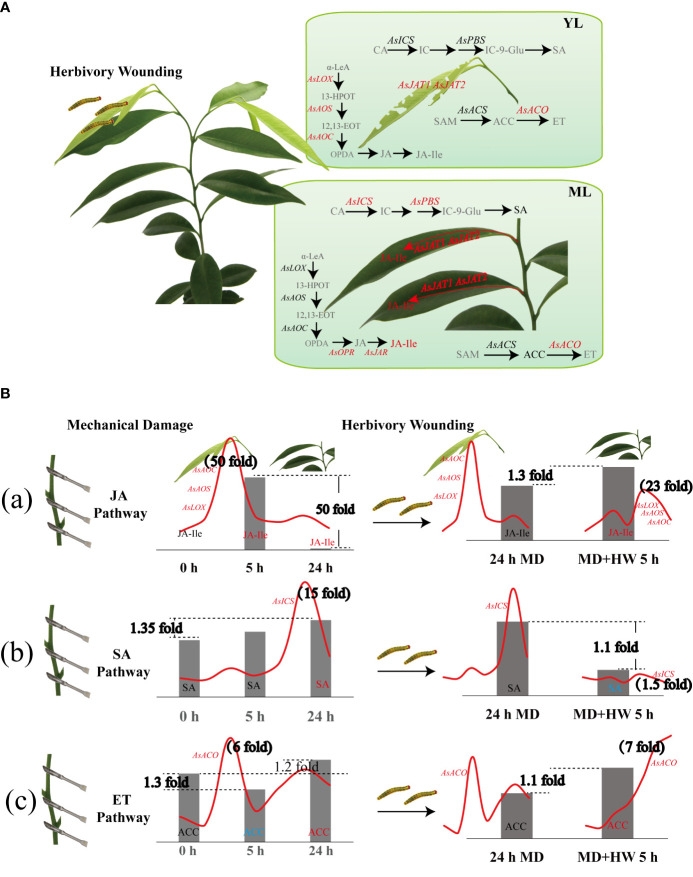
Diagram showing the variation of phytohormone pathways in *A. sinensis* in response to HW, MD, and the combination of HW and MD (MH). **(A)** Effect of HW on JA, SA, and ET biosynthesis. Gray fonts represent metabolites that were not examined. Red font, metabolites with elevated contents or upregulated genes. Black fonts, metabolites and genes with no difference in content or level. Arrows represent positively regulated metabolite biosynthesis. **(B)** Effect of MD on the *A. sinensis* response to HW. (a) Variation of JA-related gene expression and JA-Ile content in YL and ML. (b) Variation of SA-related gene expression and SA content in YL and ML. (c) Variation of ET-related gene expression and ACC content in YL and ML. The red curves represent the expression patterns of key genes involved in JA, SA, and ET biosynthesis over 0–24 h after the different treatments. Metabolite contents under the different treatments are shown as bar graphs. Peak values of gene expression and metabolite fold-change are indicated in bold black font. α-LeA, linolenic acid; 13-HPOT, 13-hydroxyperoxy-octadecadi(tri)enoic acid; 12,13-EOT, 12,13(S)-epoxy-octadecaenoic acid; OPDA (12-oxophytodienoic acid); OPR, 12-oxo-phytodienoic acid reductase; JA, jasmonate; JAR: JASMONOYL ISOLEUCINE CONJUGATE SYNTHASE; JA-Ile, jasmonyl-isoleucine; JAT, jasmonate transporter; CA, chorismic acid; IC, isochorismate; ICS, isochorismate synthase; PBS, avrPphB susceptible; IC-9-Glu, IC-9-glutamate; SA, salicylic acid; SAM, S-adenosyl-methionine; ACS, 1-aminocyclopropane-1-carboxylic acid synethetase; ACC, 1-aminocyclopropane-1-carboxylic acid; ACO, 1-aminocyclopropane-1-carboxylic acid oxidase; ET, ethylene.

Taken together, our findings reveal the phytohormone pathways used by *A. sinensis* in its response to HW and MD (**Figure 8**). Upon HW, damaged local YL initiate JA biosynthesis, displaying a dramatic upregulation of *AsLOX*, *AsAOS*, and *AsAOC* expression. Whereas in ML the three genes expression were much lower than putative *AsOPR, AsJAR, AsJATs* genes. Compared to the JA pathway, the genes involved in SA and ET biosynthesis were expressed at a much lower level (**Figure 8A**). The mechanisms by which MD enhanced the *A. sinensis* defense against *H. vitessoides* larvae can therefore be described in two steps. First, MD induced the expression of JA-related genes, promoting JA-Ile production at an early stage (2 h). JA-Ile content decreased later (after around 24 h), however. When MD-treated plants were exposed to herbivory, JA biosynthesis was activated once again (**Figure 8B-a**). *AsICS* expression and SA accumulation occur in the later phase of the MD response (12 h to 24 h and beyond), but is limited by HW (**Figure 8B–b**). The ET pathway exhibits a quite complex pattern under MH treatment. After MD treatment, ACC content decreased at 5 h and increased at 24 h. Upon HW, ACC increased at 5 h, with a gradual upregulation of *AsACO* expression (**Figure 8B–c**). In short, JA, SA, ET pathway are involving in *A. sinensis* response to HW, MD, and MH. JA pathway plays a dominant role in insects resistance enhanced by MD.

## Data availability statement

The original contributions presented in the study are included in the article/**Supplementary Material.** Further inquiries can be directed to the corresponding authors.

## Ethics statement

Ethical review and approval was not required for this study on animals in accordance with the local legislation and institutional requirements.

## Author contributions

The research project was designed by YC, KL, and RJ. YC, SL, and SW performed most of the experiments. BL, KW, and YZ cultured *H vitessoides* larvae and performed the growth experiments. XH partially contributed to project design and data analysis. RY and ZY collected *H vitessoides* eggs and *A sinensis* seeds and cultured *A sinensis* saplings. YC wrote the original draft of the manuscript. KL, RJ, and YS edited the manuscript. All authors contributed to the article and approved the submitted version.
