# Supplementary material for: Repeated mechanical damage enhanced Aquilaria sinensis resistance to Heortia vitessoides through jasmonic acid
Source: Front Plant Sci. 2023 Aug 8;14:1183002. doi: 10.3389/fpls.2023.1183002 (PMC10442551; doi:10.3389/fpls.2023.1183002)
Supplement: Supplementary file 1 [file DataSheet_1.docx]

Supplementary Figures

Title : Repeated Mechanical Damage Enhanced *Aquilaria sinensis* Resistance to *Heortia vitessoides* through Jasmonic Acid

**Figure S1**


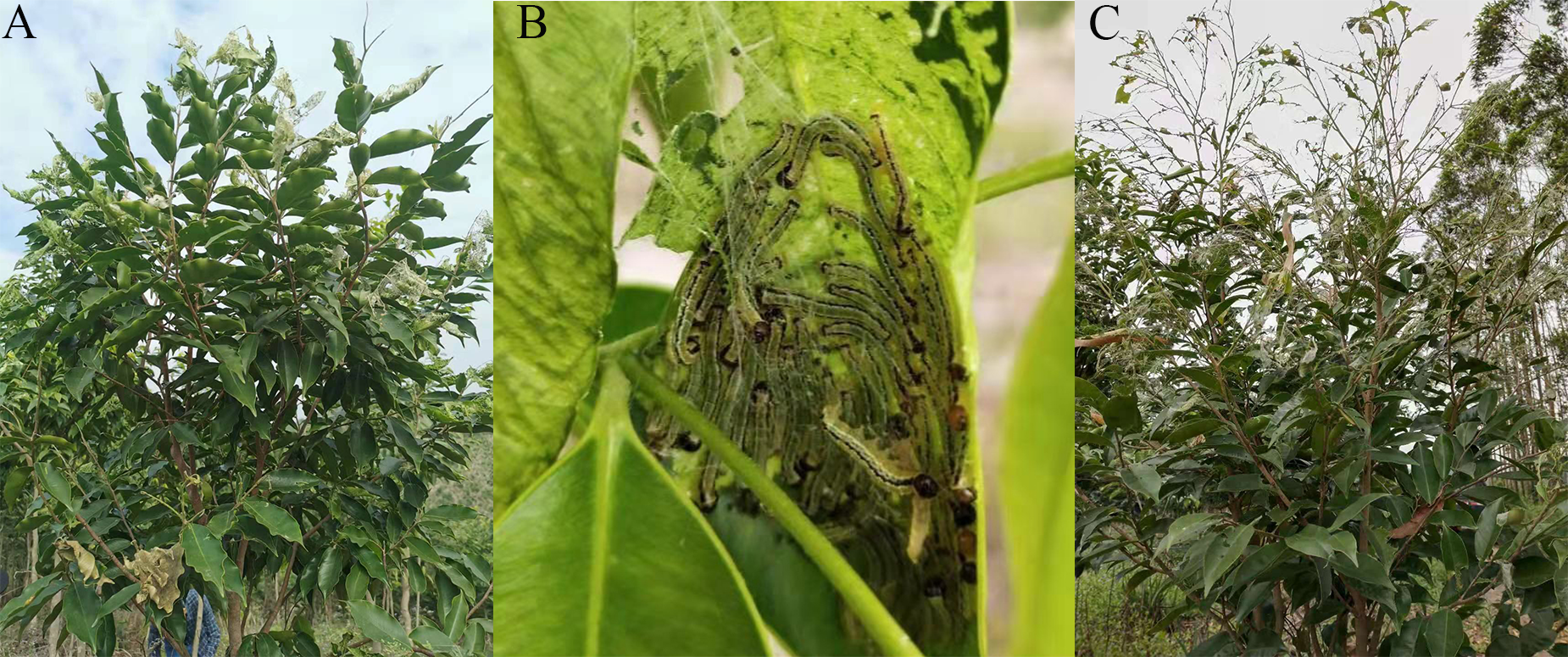


Figure S1. The phenotype of *A. sinensis* fed by *H. vitessoides* larvae in nature. (A) Most of young leaf were fed by larvae. (B) The larvae gather together to feed. (C) Mature leaf were severely consumed by larvae.

**Figure S2**





Figure S2. Effect of mechanical damage on *H. vitessoides* larvae feeding performance. The numbers labeled in column indicate the percentages of larvae that chose control or MD plants. I.S. and the below number represent the invalid selection percentage. Asterisks indicate significant differences (*, *P* < 0.05; or **, *P* < 0.01; Student’s t-test). Data are means ± SE (n ≥ 100). Three replicates were conducted.

**Figure S3**

**Figure S3.** Effect of MD on the recovery ability of *A. sinensis* saplings after *H. vitessoides* larvae feeding. (A) Survival rate of control and MD plants after larval feeding. (B) Representative phenotypes of control and MD *A. sinensis* saplings predated by larvae for four months. Asterisks indicate significant differences (*, *P* < 0.05; Student’s *t*-test). Data are means ± SE (n = 15). Three replicates were performed.

**Figure S4**


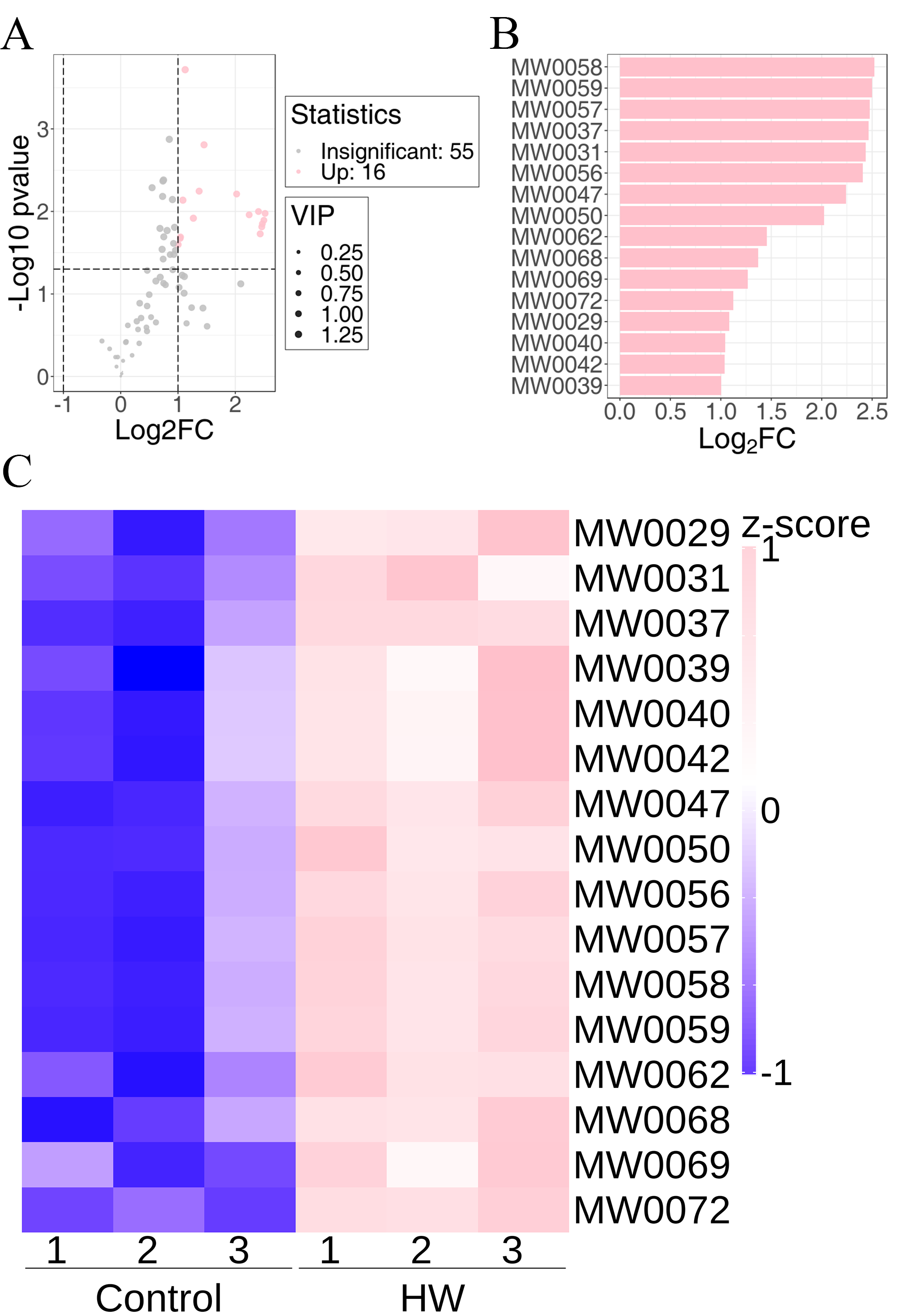


**Figure S4.** Hierarchical clustering and correlation analysis of differentially abundant metabolites in mature leaves of *A. sinensis* following HW.

**Figure S5**


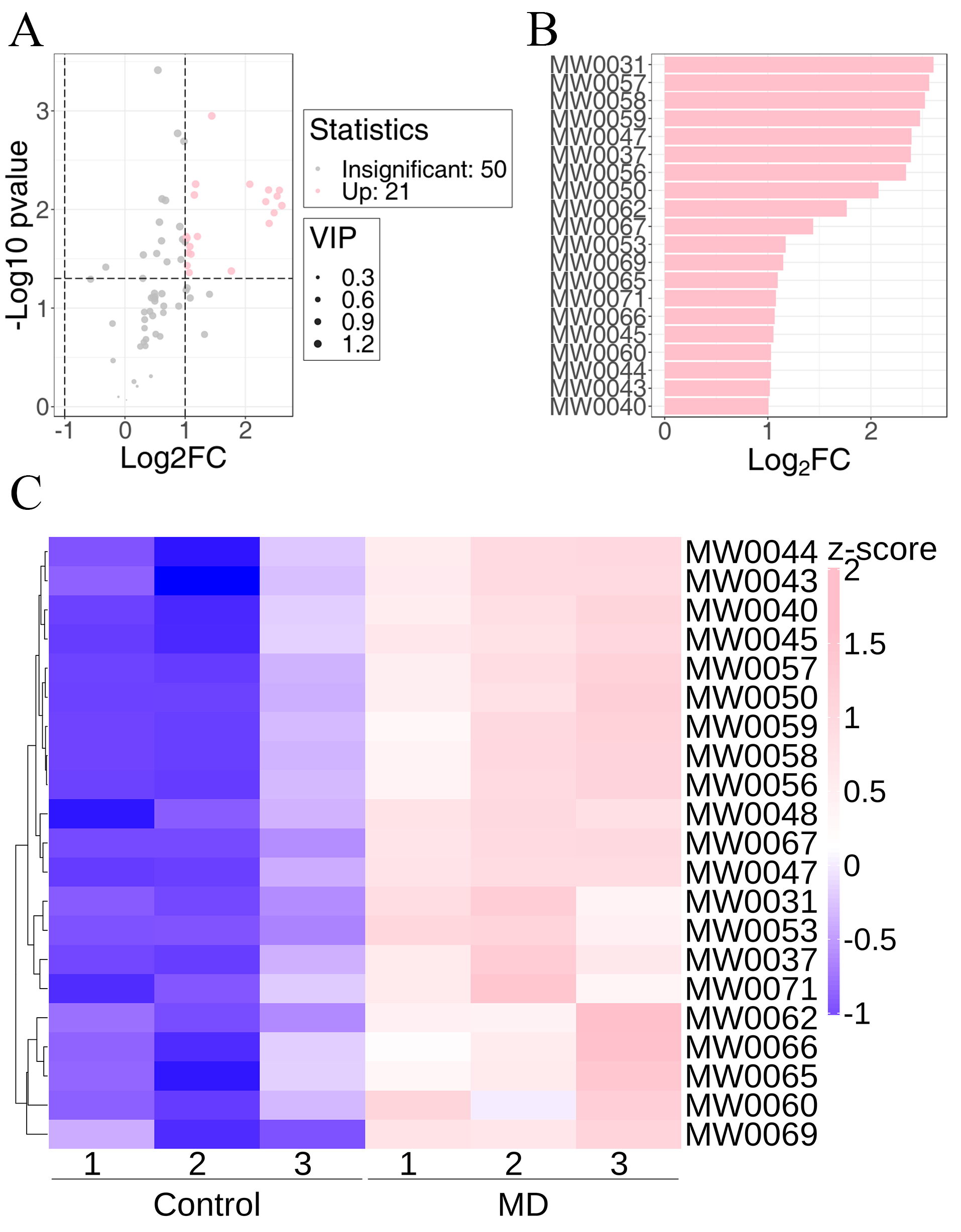


Figure S5: Hierarchical clustering and correlation analysis of differentially abundant metabolites in ML of A. sinensis following MD.

**Figure S6**


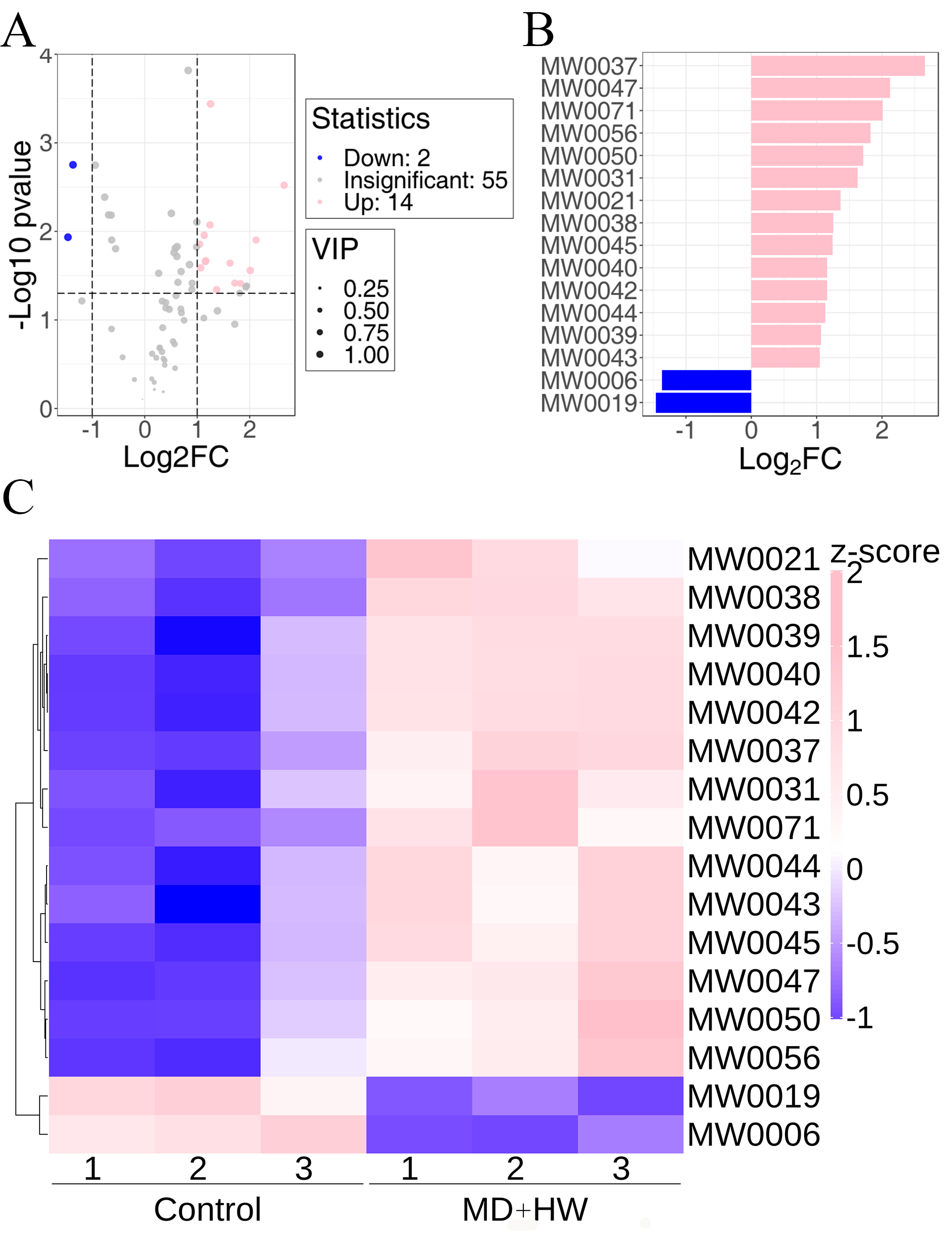


Figure S6: Hierarchical clustering and correlation analysis of differentially abundant metabolites in ML of *A. sinensis* following MH.

**Figure S7**





Figure S7. Effect of phenitone on *H. vitessoides* larvae feeding performance. Four-month-old saplings were pretreated with 0.5 mM phnitone (Phe). After 12 h, these saplings were damaged by scalpel (MD). Observed the preference of one-week-old larvae to control (without any treatment) and phe+MD (PM) plants. The numbers labeled in column indicate the percentages of larvae that chose control or MD plants. I.S. and the below number represent the invalid selection percentage. Asterisks indicate significant differences (*, *P* < 0.05; or **, *P* < 0.01; Student’s t-test). Data are means ± SE (n ≥ 100). Three replicates were conducted.

**Figure S8**


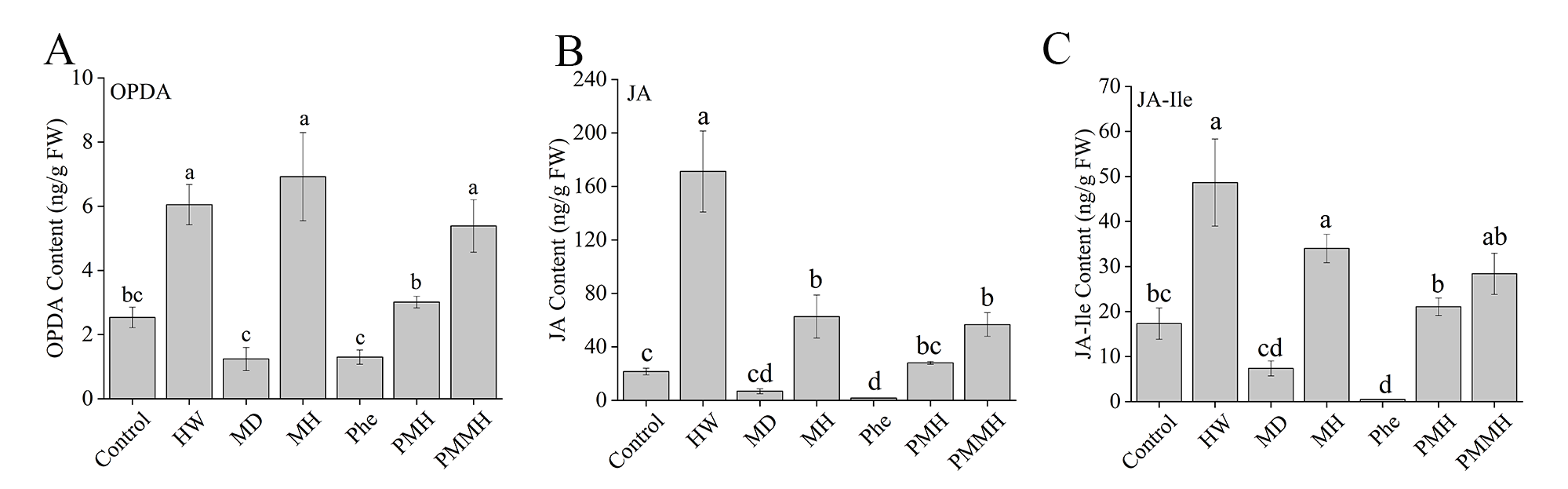


Figure S8. Effect of phenitone and MeJA on endogenous jasmonates compounds content. Ten-month-old saplings were treated with HW (herbivory wounding for 5 h), MD (mechanical damage for 24h), MH (MD-24h following HW-5h), Phe (0.5mM phenitone for 12 h), PMH (combination of Phe and MH), PMMH (Phe+MeJA+MH), respectively. (A)The variation of OPDA content in A. sinensis leaves under different treatments. (B) JA content in A. sinensis leaves under different treatments. (C) JA-Ile content in A. sinensis leaves under different treatments. Each column value represents the mean±SE (n = 3). Different letters indicate significantly different values at *P* < 0.05.
