# Supplementary material for: Repeated mechanical damage enhanced Aquilaria sinensis resistance to Heortia vitessoides through jasmonic acid
Source: Front Plant Sci. 2023 Aug 8;14:1183002. doi: 10.3389/fpls.2023.1183002 (PMC10442551; doi:10.3389/fpls.2023.1183002)
Supplement: Supplementary file 4 [file Table_3.docx]

**Complex Mechanisms in the *Aquilaria senensis* Response to Repeated Mechanical Damage and Herbivore Wounding**

**Table S3.** Aphicidal activity of fenobucarb against *H. vitessoides* larvae. LT_50_ values indicate the time to 50% mortality. All data represent the mean of five replicates.

| **Treatment** | **Concentration of fenobucarb (g/mL)** | **LT_50_ (min）** | **Larvae Phenotype** |
| --- | --- | --- | --- |
| H_2_O | 0 | 0 | 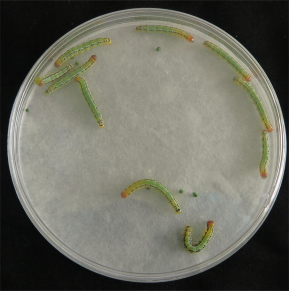 |
| fenobucarb  pesticide | 0.16 | 120±7.97a | 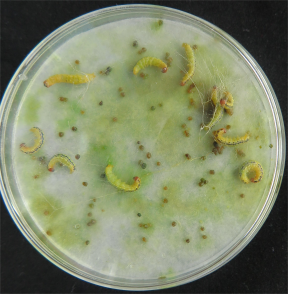 |
|  | 0.08 | 150±7.87b | 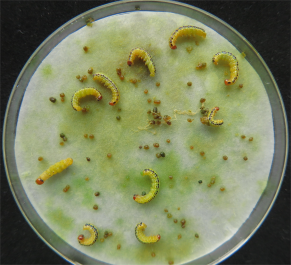 |
|  | 0.05 | 209.2±7.85c | 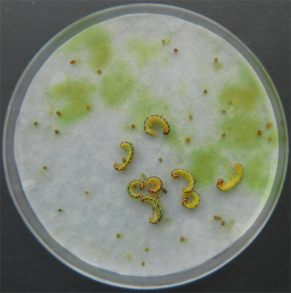 |
